# Pervious Concrete as a Controlled Stormwater Capture–Pretreatment Interface in a School-Scale Decentralized Harvesting System

**DOI:** 10.3390/ma19102129

**Published:** 2026-05-19

**Authors:** Roberto Fernando Frausto Castillo, José de Jesús Pérez Bueno, Pablo Osiris Rodríguez Zamora, Horacio Tinoco Montañez, José Alfredo Ramírez Guerrero, Ma. de Lourdes Montoya García, Ángel López Jiménez, Carlos Estrada Arteaga, José Luis Reyes Araiza, Maria Luisa Mendoza López, Alejandro Manzano-Ramírez

**Affiliations:** 1 Centro de Investigación y Desarrollo Tecnológico en Electroquímica, S.C., Parque Tecnológico Querétaro-Sanfandila s/n, Pedro Escobedo 76703, Qro, Mexico; jramirez@cideteq.mx (J.A.R.G.); lmontoya2908@yahoo.com.mx (M.d.L.M.G.); alopez@cideteq.mx (Á.L.J.); cestrada@cideteq.mx (C.E.A.); 2 Cetis 105, Carretera, A Tlacote, El Bajo, Santiago de Querétaro 76137, Qro, Mexico; pablo.rodriguez@cetis105.edu.mx (P.O.R.Z.); horacio.tinoco@cetis105.edu.mx (H.T.M.); 3 Universidad Autónoma de Querétaro, Facultad de Ingeniería, Cerro de las Campanas s/n, C.P. 76010, Cto. Universitario, Centro Universitario, Santiago de Querétaro 76010, Qro, Mexico; araiza@uaq.edu.mx; 4 Tecnológico Nacional de México, Instituto Tecnológico de Querétaro, Av. Tecnológico s/n Esq. M. Escobedo Col. Centro, Santiago de Querétaro 76000, Qro, Mexico; maria.ml@queretaro.tecnm.mx; 5 Centro de Investigación y de Estudios Avanzados del I.P.N., Unidad Querétaro, Libramiento Norponiente #2000, Fracc. Real de Juriquilla, Santiago de Querétaro 76230, Qro, Mexico; amanzano@cinvestav.mx

**Keywords:** stormwater harvesting, pervious concrete, controlled conveyance, staged pretreatment, decentralized water reuse, sand/gravel filtration, activated carbon, educational prototype

## Abstract

Urban stormwater is often viewed as a drainage problem rather than a local water resource, even in areas where runoff capture could simultaneously reduce flooding and promote the reuse of non-potable water. This study develops, installs, and field-tests a decentralized, school-scale stormwater harvesting system that relocates permeable concrete, transforming it from a passive infiltration surface into a purpose-built capture and pretreatment interface. The system integrates a 3 m × 3 m permeable concrete slab with load-bearing sections, an impermeable underlayer to ensure controlled flow, a double-compartment sump for staged sedimentation and hydraulic damping, sequential filtration with sand/gravel and activated carbon, and a 5000 L storage tank. The prototype was implemented at CETis 105 in Querétaro, Mexico, and evaluated during its commissioning and operation in the 2023 rainy season. Field operations demonstrated reduced ponding in the catchment area and a reliable flow of runoff to the pretreatment units. In the sump compartments, apparent color decreased from 221 to 59 Pt-Co, turbidity from 46.8 to 12.9 NTU, and COD from approximately 30–35 to 15–18 mg·L^−1^, corresponding to approximate pretreatment reductions of 73.3%, 72.4%, and 40–57%, respectively, before post-filtration. Conversely, the elevated pH, electrical conductivity, and total dissolved solids indicated interaction with fresh cementitious materials and dissolved ionic residues during initial operation, highlighting the need for curing, initial washing, and post-filtration verification before declaring compliance with reuse requirements. Therefore, the results support the feasibility of the proposed configuration as a decentralized, low-infrastructure architecture for localized runoff control and pretreatment, while confirming that full reuse validation still requires microbiological and post-filtration evaluation. The study provides a field-proven system design adaptable to school campuses and similar institutional environments for distributed stormwater management and non-potable water storage.

## 1. Introduction

Urban areas face a dual, increasingly synchronized challenge: pluvial flooding driven by intense rainfall and land sealing, and growing pressure on potable water supplies due to climate variability, rapid land-use change, and rising demand. This pressure is not only quantitative but also qualitative, as contamination and non-point-source pollution continue to degrade surface and groundwater, undermining the resilience of both communities and biodiversity. A defining paradox of modern cities is that they can suffer water stress while simultaneously expelling large volumes of rainwater as nuisance runoff. This disconnection is especially evident in Latin American contexts, where sustainable urban drainage systems (SUDS) and water-sensitive approaches are increasingly framed as essential pathways for climate adaptation [1]. Stormwater harvesting and reuse are therefore shifting from being treated solely as drainage solutions to being recognized as strategic drought-buffering strategies when capture, treatment, and storage are integrated into the built environment. Recent reviews on stormwater harvesting emphasize that implementation is increasingly driven not only by flood mitigation, but also by water scarcity, climate adaptation, distributed storage, and fit-for-purpose non-potable reuse. This shift has moved the field toward integrated systems in which runoff capture, pretreatment, storage, and end-use quality criteria are evaluated together rather than as isolated design problems [2,3,4]. The scientific debate has moved from whether runoff can be used to how to harvest and treat it safely, economically, and at scale [2]. Beyond the infiltration benefits, permeable pavements have also been integrated into stormwater collection treatment trains, where collection, pretreatment, and downstream filtration/storage are intentionally arranged for reuse [3,4].

Within SUDS, low impact development (LID), and water sensitive urban design (WSUD), permeable pavements have become key technologies because they attenuate runoff peaks, support distributed infiltration or controlled conveyance, and can contribute to pollutant reduction, with bibliometric evidence confirming the rapid expansion of the field across both hydraulic and water-quality functions [5]. Among these options, pervious concrete stands out for its open-graded structure and high interconnected porosity, enabling rapid infiltration and short-term storage within the pavement voids [6].

Pervious concrete is conventionally described as a near-zero-slump, open-graded cementitious composite composed mainly of coarse aggregate, cementitious binder, water, and admixtures, with limited or no fine aggregate [7,8,9]. This formulation produces a highly interconnected void structure, which is the defining microstructural feature of the material and the principal determinant of its coupled permeability–strength behavior [10,11,12]. Across the literature, typical void contents are reported in the range of 15–35%, whereas compressive strengths are frequently reported in the range of 2.8–28 MPa [7,8,9]. Hydraulic performance is much broader because infiltration and permeability depend strongly on aggregate gradation, pore connectivity, and compactive effort or mixture density [10,11,12]. Consequently, pervious concrete can be understood as a material positioned between conventional structural concrete and purely drainage-oriented porous media, since its open-pore skeleton enables both load support and controlled stormwater conveyance [7,8,9].

The performance envelope of pervious concrete is reinforced by state-of-the-art syntheses showing that mixture proportioning, aggregate grading, paste content, and construction and curing govern the hydraulic–mechanical tradeoff, and that increasing porosity improves permeability but can reduce mechanical capacity unless the paste–aggregate interface, gradation, and compactive effort are carefully optimized [7,8,9]. More recent studies have also shifted the discussion from total porosity alone toward pore-structure-informed design, showing that connectivity, tortuosity, pore-size distribution, aggregate size, and paste distribution are decisive for predicting permeability and balancing hydraulic performance with strength. This is relevant to the present work because the pervious concrete layer is not evaluated solely as a pavement material, but as a hydraulic interface whose performance depends on maintaining connected flow paths while preserving sufficient local mechanical stability [10,11,12,13,14,15].

From a sustainability and water-management perspective, pervious concrete has also been evaluated as a permeable pavement solution for runoff attenuation, pollutant reduction, and urban cooling. However, recent reviews emphasize that its greatest value lies in its integration into an urban water-cycle strategy rather than in its treatment as a standalone pavement material [9,16,17,18].

Material innovations, including supplementary cementitious materials (SCMs)-rich binders, nanoscale modifiers, recycled aggregates, fibers, and geopolymer variants, have been explored to improve durability and partially recover mechanical capacity while preserving permeability [19,20,21,22]. These approaches are relevant because pervious concrete must be optimized as a coupled hydraulic–mechanical material, not only as a high-void pavement layer.

Despite these advances, the dominant barrier to long-term hydraulic performance in real installations remains clogging. Field and laboratory evidence show progressive permeability loss due to sediment accumulation, with maintenance regimes and pretreatment strategies being decisive factors in functional lifespan [23,24]. This reality reframes success metrics: initial infiltration is not the real measure of performance; retained infiltration after sediment loading and service time is. It also clarifies why pervious concrete is increasingly justified not only for drainage but for water-quality improvement when it is positioned as an upstream barrier within treatment trains rather than as a standalone purifier. A recent critical synthesis reinforces that pervious/permeable systems can reduce contaminant loads through combined physical, chemical, and biological mechanisms, but that efficiency depends on pore structure, hydraulic residence time, influent chemistry, and the composition of underlying or secondary media [25]. Empirical studies on engineered bases show improved nutrient and metal removal under controlled conditions, strengthening the role of pervious concrete as a first barrier inside a more comprehensive treatment architecture [26]. The persistence of clogging and the need to preserve filter life are consistent with the established evidence for staged solids control and low-energy polishing of particulates and dissolved organics using granular media and activated carbon in decentralized contexts [27,28]. Because clogging can alter infiltration and runoff behavior by forming bottlenecks and rapidly degrade hydraulic function, upstream solids management is essential when the goal is stable harvesting performance [29]. Recent work on contaminant removal in pervious and permeable pavement systems further supports this multi-barrier interpretation. Pollutant reduction depends on pore structure, hydraulic residence time, influent chemistry, sediment accumulation, and the composition of underlying or downstream media. Therefore, the use of a staged sump before sand/gravel and activated-carbon filtration is consistent with current evidence that pretreatment and media protection are necessary when permeable pavements are used as part of a water-quality treatment train rather than only as drainage surfaces [25,26,27,28,29].

System-level approaches that combine permeable pavements with storage tanks and complementary treatment steps appear to deliver more robust performance than single-component designs. Field evidence from combined SUDS treatment trains indicates that linking permeable pavements to stormwater tanks can improve runoff-quality management while expanding the practical reuse window [30]. These treatment-train studies are the closest comparators to the present work because they evaluate permeable pavement not only as a drainage surface, but as one element within a sequence that includes storage and water-quality control. However, the present system differs by deliberately placing the pervious concrete over an impermeable sublayer to maximize recoverable flow and by incorporating a double-compartment sump with an internal pervious-concrete wall as an upstream solids-control stage before filtration [30,31,32,33,34]. Life-cycle and techno-economic studies also suggest that stormwater harvesting through pervious pavements can be competitive when potable-water savings and co-benefits are captured within the assessment boundary [31,32], and integrative life-cycle assessment (LCA) reviews encourage context-specific evaluation rather than universal performance claims [35]. Modeling work in institutional settings further supports the plausibility of campus-scale adoption by showing substantial potential potable-water savings that depend on rainfall regime and demand profile [33]. At the same time, recent research on anti-clogging and anti-freezing strategies highlights that long-term field reliability will depend on design choices that anticipate sediment loading and climatic constraints rather than on reactive measures implemented after installation [36,37].

Overall, the recent literature supports three practical design principles for decentralized stormwater reuse: capture should occur where runoff is generated, pretreatment should precede fine filtration to protect media lifespan, and water-quality targets should be tied to realistic non-potable uses. These principles also identify a clear research gap: pervious concrete should be evaluated not only as a permeable pavement material, but as part of hybrid architectures that manage clogging, retain recoverable water, and support fit-for-purpose reuse pathways [35,36,37,38,39].

Consequently, the recent literature suggests that the next research step is not simply to increase the permeability of pervious concrete, but to integrate it into hybrid architectures that manage clogging, protect downstream media, retain recoverable water, and define fit-for-purpose reuse pathways. This is the specific gap addressed by the present school-scale system.

Compared with conventional permeable pavement applications, where pervious concrete is commonly used as a passive infiltration layer, this work deliberately decouples the capture slab from soil infiltration by placing it over an impermeable sublayer. This transforms the pervious concrete surface into a controlled collection interface that routes runoff toward a defined pretreatment node. Compared with simple collection-sump configurations, the proposed system incorporates a double-compartment pumping station with an internal pervious-concrete separation wall, creating a staged solids-control step before sand/gravel and activated-carbon filtration.

Accordingly, the novelty of this study lies in the system architecture rather than in any single component: a pervious-concrete capture slab, an impermeable conveyance layer, a staged sump pretreatment unit, a sequential filtration train, a storage tank, and a reduced-scale dissemination prototype are integrated into a school-scale harvesting configuration. This approach translates known clogging and water-quality limitations of permeable pavements into a practical multi-barrier design for decentralized runoff control and non-potable reuse in low-infrastructure institutional settings.

## 2. Materials and Methods

### 2.1. Site, Design Rationale, and System Configuration

**Figure 1:**
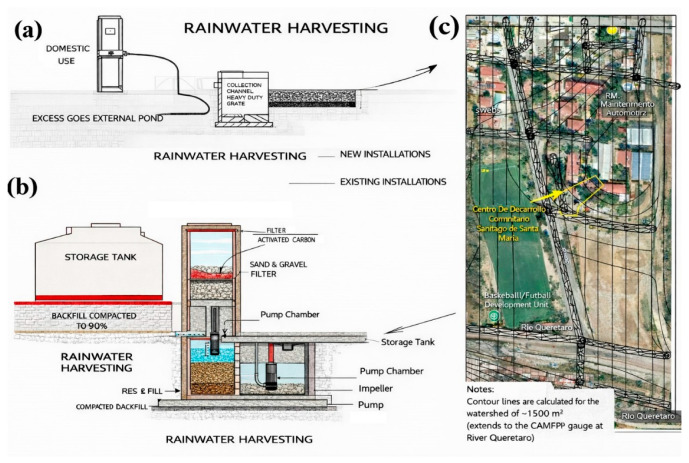
Site context and conceptual evolution of the harvesting layout: (**a**) candidate installation location within the school facilities and relative topography guiding near-source capture, (**b**) initial concept (single sump before filtration), (**c**) selected concept integrating a double-compartment pumping station with an intermediate pervious-concrete element for staged pretreatment before granular filtration. (**b**,**c**) Explicitly document the design decision used to mitigate clogging risk by adding an upstream settling/retention stage.

**Figure 2:**
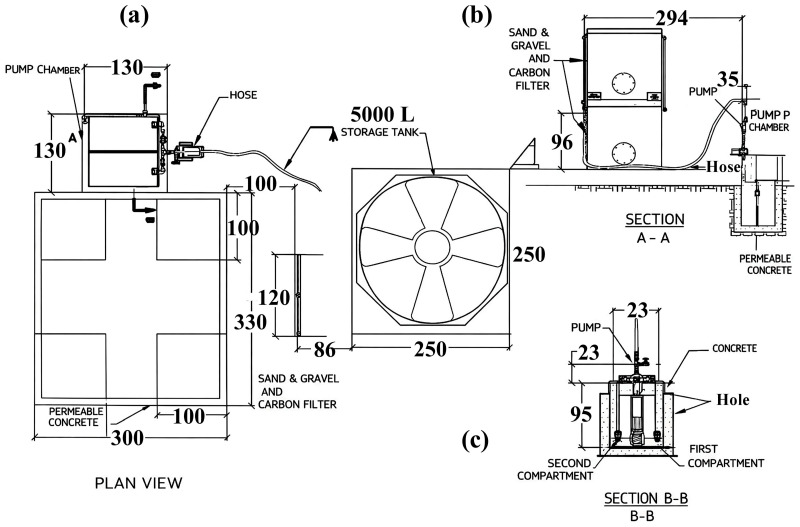
Engineering layout and flow path of the full-scale stormwater harvesting system. (**a**) Plan view of the pervious-concrete capture slab, double-compartment pumping station, filtration units, and 5000 L storage tank; (**b**) Section A-A showing the filtration units, pump connection, hose line, and pump chamber; and (**c**) Section B-B showing the double-compartment pumping station and internal pump arrangement. Dimensions are shown in cm, whereas pipe and hose labels indicate hydraulic components.

Figure 1 outlines the system architecture as a deliberate sequence of capture, pretreatment, filtration, and storage. In Figure 1a–c, the permeable slab collection interface, the lined/impermeable sublayer and sump, and the staged pretreatment units define the hydraulic logic that separates collection from uncontrolled infiltration. Figure 2 provides construction evidence of each unit and its connectivity, enabling reproducibility beyond a purely conceptual design. Table 1 compiles the key geometric and operational parameters (dimensions, materials, volumes, and flow path) used as the basis for performance interpretation and adaptation to other watersheds.

A school-scale rainwater and runoff harvesting prototype was designed and installed at CETis 105 (Santiago de Querétaro, Mexico; 20°35’31.93″ N 100°26’43.35″ W, elevation ~1804 m a.s.l.). The system targets near-source capture of on-campus stormwater, combining a pervious-concrete capture surface with controlled conveyance and a staged pretreatment–filtration pathway before storage for non-potable reuse.

The site selection considered multiple candidate locations within the campus. The facility was located at a lower elevation than the surrounding facilities to facilitate gravity-assisted collection and preserve vehicular and pedestrian access for construction, inspection, and maintenance. Clogging is a prevalent long-term failure mode in permeable pavements. Therefore, documenting staged solids pretreatment/control at the conceptual design stage is not aesthetic but functional [40].

Two conceptual versions were evaluated. The first version proposed routing the water captured by the pervious concrete slab into a simple sump before filtration. The second selected version added a double-compartment pumping station, separated by an intermediate element comprising a pervious concrete wall, enabling a second-stage pre-separation before granular filtration. This design choice explicitly shifts the system toward clogging-aware pretreatment and staged solids control. The evolution between the two versions and the site location/topography are shown in Figure 1a–c to anchor the design logic early and to justify why the second configuration is more defensible for long-term hydraulic reliability. Figure 1 frames the conceptual evolution from a simpler catchment layout to one that better aligns with staged pretreatment and clogging-aware design, providing a visual rationale that the literature often lacks when transitioning from pavement-only systems to integrated harvesting nodes.

The physical configuration of the final system comprises: (i) a 3m × 3 m pervious concrete slab; (ii) an impermeable sublayer that prevents uncontrolled infiltration and promotes controlled conveyance; (iii) a double-compartment pumping station with internal staged separation; (iv) a sand–gravel filter; (v) an activated carbon filter; and (vi) a 5000 L storage tank. This complete sequence is described as the functional backbone of the prototype. Placing the pervious slab over an impermeable sublayer aligns with the concept of lined permeable pavement reported for water quality control and discharge management, in which the pavement is used as a collection/treatment interface rather than a purely infiltrating surface [34].

### 2.2. Materials and Equipment

Construction materials and analytical reagents were procured before the commencement of civil works, and an external comprehensive construction service was contracted to execute the works.

The acquisition was carried out in stages to allow for parallel preparation: first, the analytical reagents for immediate verification of water quality were obtained, then the civil works materials and equipment, and finally, the comprehensive construction service contracted for the installation.

The installation used PVC Schedule 80 piping (½″ and 1¼″), standard hydraulic accessories (valves, elbows, unions, flanges, and hoses), a submersible pump, two in-line filter units, and a 5000 L storage tank.

### 2.3. Pervious Concrete Mixture Design and Laboratory Verification

A national commercial waterproofing additive was incorporated into the pervious concrete mixtures to reduce excessive paste porosity after setting and strengthen the aggregate–paste interfaces. Although additives with waterproofing properties can seem counterintuitive in pervious systems, their use is reported as a practical strategy for stabilizing joints between coarse aggregates in field-oriented applications. Laboratory trials were conducted to verify that the mixture remained permeable while improving the cohesion of the skeleton.

Fester^®^ Festegral^®^ (Fester/Henkel, Guadalupe, Mexico), a commercial integral waterproofing admixture for concretes and mortars, was incorporated into the pervious concrete mixtures. The product is supplied as a finely ground, light-gray powder and contains fatty-acid salts that reduce water permeability in cementitious mixtures without decreasing compressive strength. According to the manufacturer, it also allows an approximate 4–6% reduction in mixing water, improves workability, reduces bleeding, and increases durability. In this study, it was used to improve paste cohesion at aggregate contact points and reduce excessive permeability through cementitious paste bridges while preserving the connected pore structure required for hydraulic capture.

Six coarse aggregate sizes ranging from 4.75 to 19.05 mm were evaluated, with cement at 15–16.6%, coarse aggregate at 77.6–79.4%, water at ~5.3–5.8%, and additive at ~0.06–0.07% by weight, as shown in Table 1. These mixtures were prepared and tested to select a grading compatible with the structural demands of pedestrian and light-vehicle service while preserving hydraulic identity.

### 2.4. Design and Construction of the Capture Slab and Impermeable Sublayer

The capture surface consists of a 3 × 3 m pervious-concrete slab, reinforced at the corners with conventional structural concrete (compressive strength f′c ≈ 25 MPa) to support pedestrian and light-vehicle loads without compromising the catchment function.

From a structural standpoint, the capture module was designed for pedestrian and light-vehicle service rather than as a high-load pavement. The pervious concrete area provides the hydraulic capture function, while conventional structural concrete sections at the corners, with f′_c_ ≈ 25 MPa, provide localized load-bearing reinforcement. Therefore, the strength strategy of the constructed system is based on functional separation between hydraulic capture and localized structural support. Direct mechanical testing of the installed pervious slab, including core extraction, flexural testing, fatigue testing, or numerical structural simulation, was outside the scope of the present field validation and is identified as a necessary future step for broader pavement-design certification.

A key experimental decision was to place the pervious slab over an impermeable layer. This ensures that the system behaves as a controlled-conveyance collector rather than a pure infiltration pavement, enabling consistent routing of runoff toward the pumping station while minimizing losses to the subsoil. This design principle is central to this work proposal and is visually supported by the cross-section details in Figure 2a. This stepped architecture aims to protect the downstream media from sediment loading and delay functional clogging [41].

### 2.5. Double-Compartment Pumping Station

The pumping station functions as a double-compartment sump for staged pretreatment. A physical divider creates hydraulic buffering and promotes sedimentation; in the selected design, a pervious-concrete internal wall at the overflow point provides an additional solids-retention stage before the filtration train. This unit, therefore, operates as a pre-decantation and buffering node rather than a simple collection sump.

### 2.6. Filtration Train and Storage

After pretreatment in the pumping station, water is routed through two sequential filters:

1. Sand and gravel filtration;
2. Activated carbon filtration, before reaching the 5000 L storage tank.

Downstream treatment consists of two readily available packed-bed units: (i) a sand/gravel filter for particulate retention and residual turbidity control, followed by (ii) an activated-carbon filter intended to reduce color and dissolved organics. Both filter housings were coupled to a 5000 L storage tank; a 2″ overflow line was included as a safety bypass to prevent overfilling.

### 2.7. Operation and Functional Sequence of the Constructed System

The constructed system operates as a controlled stormwater capture and conveyance unit in which runoff is intercepted at the surface by the pervious concrete slab and subsequently redirected through a lined hydraulic pathway. Unlike conventional permeable pavement systems designed primarily for infiltration, the present configuration incorporates an impermeable sublayer beneath the pervious concrete, preventing uncontrolled percolation into the subsoil and forcing captured water to be conveyed toward the collection and treatment units. This design transforms the pervious concrete slab into a predictable hydraulic interface for runoff harvesting rather than a loss-dominated infiltration medium.

Once the runoff passes through the pervious concrete layer, it is directed by the impermeable sublayer toward the double-compartment pumping station, where the first stage of treatment occurs. The initial compartment serves as a primary sedimentation and hydraulic-buffering zone, allowing coarse particles and suspended solids to settle under reduced flow conditions. The second compartment receives partially clarified water through an elevated transfer pathway that incorporates an internal pervious-concrete separation wall. This intermediate element provides an additional stage of solids retention, promoting further reduction in particulate load before the water is subjected to mechanical conveyance. In this configuration, the pumping station operates not only as a hydraulic node but also as a staged pretreatment unit designed to mitigate clogging and protect downstream filtration systems.

Following the rainfall event, the system is operated manually, activating the pump to extract water from the second compartment. This operational strategy ensures that the withdrawn water has undergone preliminary clarification, reducing the transport of suspended solids into subsequent treatment stages. The pumped flow is then directed sequentially through a granular filtration unit composed of sand and gravel, followed by an activated carbon filter. The granular filter primarily removes residual suspended particles and turbidity, while the activated carbon unit contributes to the adsorption of dissolved organic compounds, odors, and other contaminants.

After filtration, the treated water is conveyed to a 5000 L storage tank, where it is retained for non-potable applications, such as irrigation or controlled campus use. The system is designed to operate in discrete cycles associated with rainfall events, separating the processes of capture, pretreatment, and final conditioning in both time and space. This temporal decoupling enables greater operational control, facilitates maintenance, and reduces the risk of overloading downstream treatment units during peak runoff.

From an engineering perspective, the overall configuration establishes a defined multi-stage treatment pathway consisting of: (i) surface capture through the pervious concrete slab, (ii) controlled conveyance via the impermeable sublayer, (iii) staged pretreatment in the double-compartment pumping station, (iv) sequential filtration using granular and adsorptive media, and (v) storage for reuse. This arrangement enables the system to function as a decentralized stormwater harvesting unit, integrating hydraulic control and water-quality conditioning within a single infrastructure element.

It is important to note that the performance of the system at this stage primarily reflects the effectiveness of the capture and pretreatment components. The water-quality parameters measured in the pumping station compartments correspond to intermediate stages of treatment prior to final filtration. Therefore, these values should be interpreted as indicators of pretreatment efficiency and hydraulic conditioning, rather than as definitive metrics of water quality for reuse. Full validation of reuse suitability requires evaluation of post-filtration water quality, including microbiological assessment, which is beyond the scope of the present section.

The hydraulic description available for this field campaign is therefore based on system geometry and operational sequence rather than continuous hydraulic instrumentation. The principal hydraulic descriptors are the 3 × 3 m capture slab, the impermeable conveyance layer, the double-compartment sump, manual post-event pumping, sequential packed-bed filtration, and the 5000 L storage tank. Event-scale parameters, such as rainfall intensity, inflow hydrograph, captured volume per event, residence time in each compartment, and filter-specific flow rate, were not continuously recorded during this first field campaign. These variables should be incorporated in future monitoring to develop a complete hydraulic performance model of the system.

### 2.8. Field Testing, Sampling, and Physicochemical Analysis

Field validation was performed during the 2023 rainy season. A reference sample was collected from a puddle upstream of the system on 25 October 2023 to represent incoming stormwater/runoff before treatment. Two additional samples were collected from the pumping station compartments to evaluate pretreatment performance and to confirm the need for granular and activated carbon filtration. Samples were refrigerated until analysis.

The evaluation uses NOM-003-SEMARNAT-1997 as a fit-for-purpose benchmark for reclaimed water intended for uses involving controlled or occasional human contact (e.g., irrigation and similar non-potable scenarios). In this study, sampling focused on influent reference water (a puddle upstream of the system) and on the two sump compartments before filtration, to quantify pretreatment gradients and identify constraints requiring downstream polishing.

For final non-potable reuse, the sand/gravel and activated-carbon units should be interpreted as polishing stages that reduce residual suspended solids, color, and dissolved organic constituents, but not as standalone microbiological safety barriers. Therefore, reuse applications, such as toilet flushing, irrigation, or controlled campus use, should be validated using post-filtration samples collected at the storage-tank outlet. Minimum safety monitoring should include total coliforms and *Escherichia coli*, and applications involving possible human contact or aerosol formation should include a disinfection barrier, such as chlorination or UV treatment, together with periodic cleaning of the storage tank. These recommendations are necessary before moving from pretreatment validation to fit-for-purpose reuse authorization.

### 2.9. Maintenance Strategy and Consumable Replacement Criteria

Maintenance was defined as an operational requirement of the decentralized harvesting system because solids accumulation, pump reliability, and filter-media saturation determine long-term functionality (Table 2). The system is manually operated; therefore, inspection and maintenance must be linked to rainfall events and observed operating conditions rather than only to fixed calendar intervals. After rainfall events, the pervious concrete surface should be inspected and cleared of leaves, trash, and coarse sediment to reduce surface clogging. The sump compartments should be inspected for accumulated sediment and standing water, and sediment should be removed when deposits become visible or hydraulic transfer between compartments decreases.

The pump and hydraulic lines should be inspected during operation to verify suction, discharge, valve position, and absence of leaks. If the pump remains inactive for extended periods, flush the line with clean water before restarting. The sand/gravel filter should be backwashed or cleaned when turbidity breakthrough, flow reduction, or visible solids accumulation is observed. If backwashing does not restore flow or clarification capacity, the granular medium should be replaced. The activated-carbon unit should be considered a consumable polishing stage because its adsorption capacity decreases as dissolved organic compounds and other contaminants accumulate. Under continuous use, replacement or regeneration of the activated carbon is recommended approximately every 2 years, although the actual interval should be adjusted based on treated volume, influent quality, and breakthrough indicators such as color, odor, or COD increases.

From a cost perspective, the recurrent maintenance burden is expected to be dominated by manual labor for surface cleaning and sump sediment removal, periodic backwashing or replacement of sand/gravel media, pump inspection, and activated-carbon replacement or regeneration. Because labor rates, material prices, rainfall frequency, and sediment loading vary by location, the present study does not assign a universal cleaning cost. Instead, the maintenance framework identifies the main cost drivers and operational triggers, enabling future implementations to calculate local maintenance costs using site-specific labor and material prices.

## 3. Results and Discussion

### 3.1. System Implementation, Construction Sequence, and Commissioning

To ensure the reproducibility of the site selection, the design was limited by (i) the local topography, which guarantees gravity transport to the pretreatment train, (ii) safe access for construction and maintenance, and (iii) proximity to the educational center to facilitate long-term monitoring. Therefore, Figure 2a,b (site and design views) function as a map of design constraints, rather than a simple photographic record.

#### 3.1.1. Site Selection and Layout Logic

The installation site was selected within the school campus to exploit the natural topographic gradient and favor passive convergence of runoff toward the capture zone. The lowest-elevation area was prioritized to maximize collection efficiency during high-intensity rainfall events while minimizing the need for complex ancillary conveyance structures. This decision also aligned with the system’s dual purpose: reducing recurrent waterlogging and channeling a portion of stormwater into a controlled treatment and storage route for non-potable reuse. The layout was therefore designed not only as a pavement intervention but also as a localized drainage extension strategy that intercepts runoff from both paved and dirt surfaces, thereby reducing surface accumulation during rainy periods.

**Figure 3:**
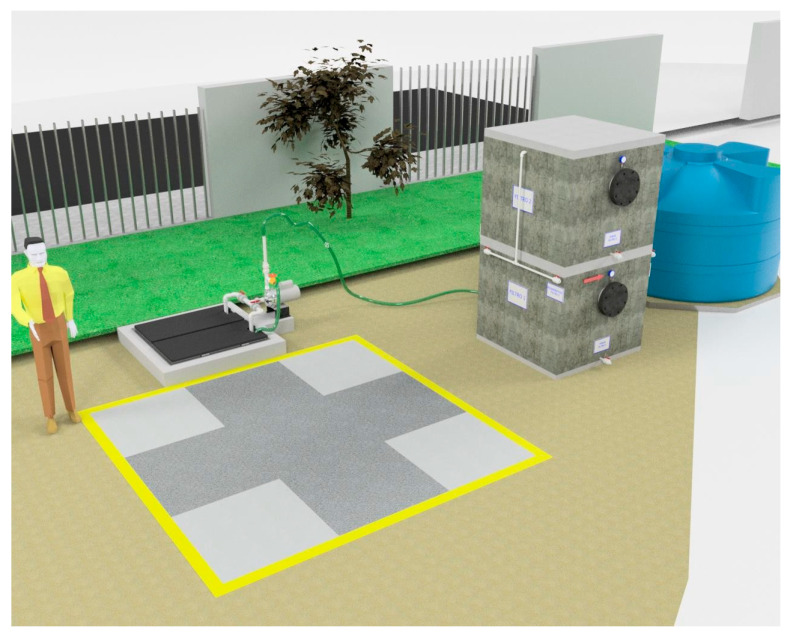
External visualization and location of the system installed within the school grounds, showing the surrounding drainage context that necessitates near-source catchment and the practical accessibility limitations for construction and maintenance.

**Figure 4:**
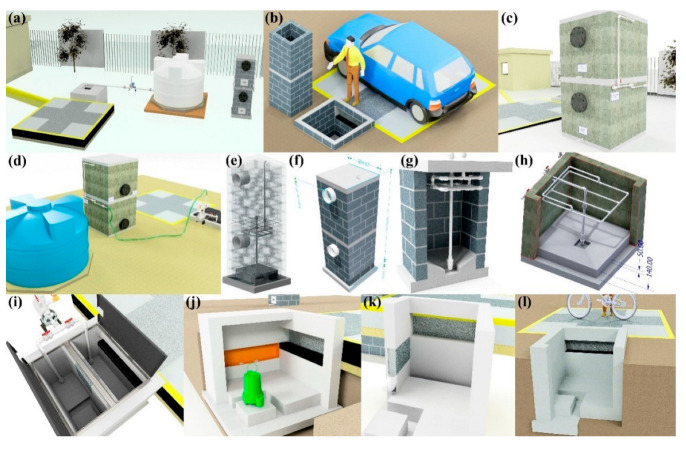
Modeled views of the permeable concrete rainwater harvesting system, detailing the key functional elements: (**a**) overview of the system integrated into the school environment, (**b**) detail of the surface catchment, (**c**) double-compartment pumping sump, (**d**) connection to the 5000 L storage tank, (**e**,**f**) structural cuts of the sump that define the pre-separation zones and the pumping elements, (**g**,**h**) internal distribution of filter media of sand, gravel and activated carbon, (**i**,**j**) functional models of hydraulic operation, and (**k**,**l**) application of permeable concrete in pedestrian and light vehicle crossing areas.

The construction sequence is considered part of the scientific contribution, as it determines the hydraulic integrity and water quality outcomes. Figure 3 documents the construction of the capture slab and the installation of the liner (including the sealing interfaces), while Figure 4 verifies the assembled pretreatment modules and their coupling (sump/settling tank/first filtration) before commissioning. These figures are used here as quality control points (minimizing leakage paths, ensuring proper stage connectivity, and facilitating easy maintenance access), rather than simply as illustrative photographs.

The spatial arrangement of the capture slab, pumping station, filtration train, and storage tank was defined to create a short, inspectable hydraulic pathway that can be implemented under school-level infrastructure constraints. This compact configuration reflects the intended replicability of the system in similar institutional settings where budgets, maintenance capacity, and technical personnel may be limited.

#### 3.1.2. Civil Works and Installation of Capture and Pretreatment Units

The construction site context and ready-to-build geometry are documented in Figure 3 and Figure 4: Figure 3 situates the intervention within the school grounds, while Figure 4 provides modeled views of the catchment slab, the double-compartment sump, and the filtration-storage system to facilitate replication and clarify the pretreatment logic.

The filtration unit repair is explicitly reported because it limits the operational robustness of low-cost harvesting systems. Reporting this intervention provides a practical basis for interpreting any residual turbidity/COD as performance-limited by media condition, bypass paths, or solids penetration, and defines the minimum maintenance actions required for stable operation.

Civil works began with site marking, controlled excavation, and subgrade preparation to ensure stable support for the capture slab and reliable alignment of the downstream pretreatment infrastructure. The pervious concrete capture surface was cast as a defined 3 × 3 m module and structurally reinforced where necessary using load-bearing concrete sections to maintain safe pedestrian and light-vehicle functionality without compromising hydraulic capture.

A central experimental decision was to incorporate an impermeable sublayer beneath the pervious slab. Rather than allowing uncontrolled infiltration into the subsoil, this layer forces collected water to move laterally toward the intake, thereby converting the slab from a conventional infiltration pavement into an engineered capture interface. The double-compartment pumping station was then installed as the first dedicated pretreatment node, designed to buffer inflow surges and enable staged solids settling before fine filtration. The internal compartment logic, including the integration of a pervious concrete wall as a secondary pre-separation element, was implemented to align with clogging-aware design principles and to reduce the solids burden reaching the sand/gravel and activated carbon units.

#### 3.1.3. Filter-Unit Repair and Final Commissioning

Initial system startup and hydraulic testing revealed leakage in the early filtration units. This was treated as a relevant commissioning outcome rather than a minor defect, since it clarified the practical requirements for durable, low-infrastructure deployment in real school environments. The filtration bodies were therefore replaced or reinforced using stainless steel tanks to improve structural integrity and sealing reliability. After these corrections, the hydraulic circuit was re-evaluated to confirm stable flow through the pretreatment compartments and the sequential sand/gravel and activated carbon stages.

This repair-and-retest sequence is methodologically important because it defines the final as-validated configuration used for field sampling during the 2023 rainy season. It also strengthens the replicability narrative by demonstrating that the system can be repaired with accessible, modular solutions without redesigning the entire architecture. The completed commissioning stage enabled subsequent water-quality assessment under real rainfall events. It supported the manuscript’s claim that the proposed capture–pretreatment–filtration model is technically viable for non-potable reuse in water-stressed institutional contexts.

### 3.2. Construction-Stage Evidence and Replicability

Table 3 summarizes the staged construction and commissioning roadmap for the system, from initial site placement to final hydraulic validation. The sequence demonstrates that the system was not installed as a single-step civil intervention but as an iterative build–test–correct process that culminated in an operational configuration suitable for field sampling during the last rains of 2023. This chronology also clarifies the coupling between infrastructure assembly and parallel laboratory preparation of pervious-concrete specimens and filter-media packing, reinforcing the methodological coherence between material selection, unit construction, and in situ validation.

### 3.3. Pretreatment Performance Inferred from Physicochemical Gradients

The influent reference sample collected upstream of the system, represented by a puddle formed before entry into the capture zone, exhibited a light brown appearance and low settleable solids. This sample represents the combined contribution of direct rainfall and surface runoff entering the system. Two additional samples collected from the first and second compartments of the double-compartment pumping station allowed for a first-order assessment of pretreatment behavior before the water was directed to the sand–gravel and activated-carbon filtration units.

As shown in Table 4, the staged sump configuration produced a pronounced reduction in apparent color from 221 Pt-Co in Compartment 1 to 59 Pt-Co in Compartment 2. This decrease is consistent with progressive settling and early clarification within the double-compartment sump. The reduction in turbidity from 46.8 to 12.91 NTU likewise supports the role of compartmentation as a physical pretreatment step. Similarly, COD decreased from approximately 30–35 mg·L^−1^ in Compartment 1 to 15–18 mg·L^−1^ in Compartment 2, indicating that the staged pretreatment also contributed to reducing the fraction of oxidizable material associated with suspended or settleable solids. Together, these gradients confirm that the sump does not operate merely as a collection chamber, but as a hydraulic-buffering and solids-control unit that protects downstream filtration media from excessive particulate loading.

Based on these compartment-level gradients, approximate pretreatment efficiencies were calculated for the parameters that were measured in both sump compartments (Table 5). Apparent color decreased by approximately 73.3%, from 221 to 59 Pt-Co, while turbidity decreased by approximately 72.4%, from 46.8 to 12.91 NTU. COD decreased from approximately 30–35 mg·L^−1^ in Compartment 1 to 15–18 mg·L^−1^ in Compartment 2, corresponding to an estimated reduction of approximately 40–57%, depending on the values selected within the reported intervals. These reductions confirm that the double-compartment sump contributed measurable pretreatment before the water reached the sand/gravel and activated-carbon filtration units.

Reductions were calculated only for parameters measured in both compartments. COD reduction is reported as an approximate interval because both compartment values were reported as ranges.

In contrast, the elevated pH, electrical conductivity, and total dissolved solids observed in the compartments indicate a substantial ionic load during early operation. The pH increased from 8.44 in the influent reference sample to 11.0 in Compartment 1 and then decreased to 9.0 in Compartment 2. Electrical conductivity increased from 0.129 mS·cm^−1^ in the influent reference sample to 7.78 mS·cm^−1^ in Compartment 1, later decreasing to 1.963 mS·cm^−1^ in Compartment 2. A similar trend was observed for total dissolved solids, which increased from 71.71 mg·L^−1^ in the influent reference sample to 3900 mg·L^−1^ in Compartment 1 and then decreased to 985 mg·L^−1^ in Compartment 2. This behavior indicates a strong interaction between the captured runoff and fresh cementitious materials, likely due to the leaching of alkaline and ionic species from newly constructed concrete components.

The pH decrease between Compartment 1 and Compartment 2 should therefore be interpreted as part of the early leaching and dilution/stabilization behavior of the newly constructed cementitious system, not as a complete durability assessment of the pervious concrete slab. Although portlandite dissolution can contribute to elevated alkalinity and may progressively reduce the buffering capacity of cementitious materials, the present field campaign did not include direct durability tests such as compressive strength after exposure, flexural strength after wetting–drying cycles, abrasion resistance, carbonation depth, mass loss, or microstructural analysis of the slab after service. Consequently, the pH trend identifies a relevant durability-monitoring requirement rather than providing sufficient evidence to quantify long-term degradation.

This trend is consistent with previous observations in cementitious and pervious concrete systems, where early contact with water can increase pH, alkalinity, and dissolved ionic content through leaching [42,43]. Therefore, these results have two practical implications. First, curing, initial flushing, and stabilization should be treated as necessary commissioning steps before long-term operation or reuse validation. Second, the downstream sand–gravel and activated-carbon filters should be interpreted mainly as barriers for residual particulates, color, and dissolved organic compounds, rather than as primary salinity-control units. If electrical conductivity and total dissolved solids remain high after the stabilization period, reuse scenarios, such as irrigation, should be screened against EC/TDS constraints or managed through dilution, additional polishing, or alternative treatment, depending on the final end use.

The results in Table 4 should also be interpreted according to the sampling position. The reported compartment values correspond to pretreatment stages upstream of final filtration; therefore, they demonstrate the effectiveness of staged physical clarification but do not represent final post-filtration water quality. In this sense, the system-level result is not full compliance with reuse at this stage, but rather confirmation that the double-compartment sump reduces the solids and oxidizable load before the packed-bed filters. This supports the design decision of placing staged storage and pretreatment upstream of the sand–gravel and activated-carbon units in a clogging-aware harvesting architecture.

To frame the potential reuse of the harvested water, the measured indicators were interpreted against non-potable reuse benchmarks commonly applied in Mexico for treated water intended for public-service applications, particularly NOM-003-SEMARNAT-1997. However, this comparison should be understood as a preliminary fit-for-purpose benchmark rather than a final compliance claim, because the standard was developed for treated wastewater and the present dataset primarily reflects pre-filtration sampling. Final validation for non-potable applications involving controlled or occasional human contact requires post-filtration monitoring and microbiological assessment.

The generalizability of the measured water-quality values should be interpreted cautiously. The reported pH, electrical conductivity, total dissolved solids, apparent color, turbidity, and COD correspond to a specific school site, early commissioning stage, local runoff characteristics, and pre-filtration sampling locations. Therefore, these numerical values should not be transferred directly to other catchments without local validation. What is more generalizable is the system logic: controlled runoff capture through pervious concrete, prevention of uncontrolled infiltration by an impermeable sublayer, staged pretreatment in a double-compartment sump, and downstream filtration before storage. Future applications should recalibrate performance expectations based on rainfall regime, sediment load, land use, traffic exposure, maintenance frequency, and the intended reuse scenario.

### 3.4. Optical Microscopy of Solids and Salt Residues: Implications for Filter Selection

**Figure 5:**
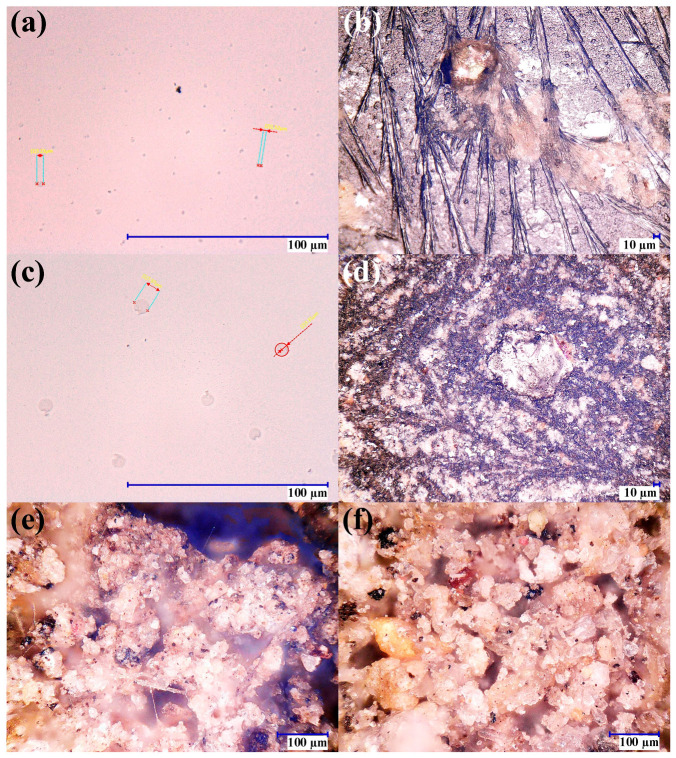
Optical micrographs of suspended solids and crystallized residues associated with harvested runoff and maintenance washings. (**a**) Fine suspended particles in sump water (2000×), (**b**) crystallized residues after drying of sump water (500×), (**c**) larger particulates in wash water collected after system cleaning (2000×), (**d**) dried residues from wash water (500×), and (**e**,**f**) irregular clay-like dried sludge particles remaining after initial pretreatment (500×).

Figure 5 provides qualitative confirmation of the contamination modes suggested by the physicochemical dataset. The observed particulate loading and ionic residues support the interpretation of turbidity/conductivity trends and justify staged solids removal (sand/gravel) followed by polishing (activated carbon). Micrographs of water from the reservoir show fine suspended particles on the order of a few micrometers, consistent with the measured turbidity in the first compartment. Drying images reveal crystallized residues, which align with elevated conductivity and dissolved solids. Washing-water micrographs exhibit larger particle fractions under pressure-cleaning conditions, while dried sludge images reveal irregular clay-like particulates spanning a wide size distribution. Together, these observations confirm that the primary early risks to hydraulic reliability and reuse suitability are particulate loading and ionic residues, validating the decision to implement sand/gravel filtration for bulk solids control followed by activated carbon as a polishing step. These observations also support the maintenance strategy summarized in Table 2, because particulate loading primarily motivates surface cleaning, sump sediment removal, and sand/gravel maintenance, whereas color and dissolved organic residues justify periodic monitoring and replacement or regeneration of the activated-carbon medium.

### 3.5. Practical Cost Positioning and Comparison with Conventional Drainage

The proposed system should be interpreted as a decentralized source-control and harvesting unit rather than as a direct replacement for a complete municipal sewer or storm-drainage network. A conventional drainage solution generally requires inlet structures, underground pipes, excavation, discharge routing, and downstream conveyance capacity, but it does not normally provide local pretreatment or storage for reuse. In contrast, the present system concentrates investment in a small capture slab, an impermeable conveyance layer, a double-compartment sump, a pump, two simple filters, and a 5000 L tank. Therefore, its practical value lies in reducing localized ponding while recovering a portion of stormwater for non-potable use.

Compared with conventional permeable pavement systems, the present configuration relies less on subsurface infiltration. Instead, the impermeable sublayer forces captured water toward a controlled treatment train, making the system more suitable for harvesting applications. Compared with lined permeable pavement systems, which are mainly used for water-quality control or discharge management, the present system adds a double-compartment sump with an internal pervious-concrete separation element to promote staged solids control before filtration. Compared with permeable pavement–storage treatment trains, the present configuration emphasizes a low-infrastructure, school-scale implementation with a didactic prototype for dissemination. Thus, the system is best understood as a hybrid between a permeable pavement, a pretreatment sump, a simple filtration train, and a decentralized storage unit.

A site-independent total cost comparison is not reported because local material prices, labor rates, excavation conditions, and drainage requirements vary widely. However, the main cost trade-off is clear: conventional drainage prioritizes rapid runoff disposal, whereas the proposed system adds local retention, pretreatment, educational value, and potential for non-potable reuse. Future implementations should include a normalized cost analysis based on the cost per square meter of treated catchment area, the cost per cubic meter of storage, and the maintenance cost per rainfall season.

Table 6 summarizes the practical comparison between the two approaches. The conventional drainage alternative is mainly cost-driven by excavation depth, pipe length, inlet structures, connection to an existing discharge route, and the hydraulic capacity required to rapidly remove runoff from the site. Its principal benefit is efficient conveyance; however, it normally does not recover water locally or provide pretreatment before discharge. In contrast, the proposed decentralized system allocates part of the investment to a capture slab, an impermeable conveyance layer, a double-compartment sump, a pump, filtration units, and a storage tank. This configuration introduces additional components that require maintenance, especially surface cleaning, sediment removal, pump inspection, filter backwashing, and activated-carbon replacement. However, these added components also provide functions that conventional drainage generally does not offer: localized runoff control, pretreatment, storage, educational visibility, and potential non-potable reuse. Therefore, the system should be evaluated not only by initial construction cost, but by the combined value of flood mitigation, water recovery, treatment function, and institutional replicability.

## 4. Design and Validation of a Prototype System for Dissemination

A didactic prototype was developed to translate the full-scale rainwater and runoff collection system installed at a school into a simplified, portable model suitable for scientific dissemination and community education. This educational extension is coherent with the manuscript’s broader aim of positioning pervious concrete as a functional harvesting–treatment node rather than a passive infiltration surface and with the demonstrated feasibility of a low-infrastructure, school-scale implementation. Site-scale feasibility and expected yields from permeable pavement harvesting depend largely on location. Spatial analyses have been used to quantify harvesting potential and to inform where such systems are practical for harvesting in significant volumes [44].

The dissemination prototype reproduces the main operational sequence of the installed system, allowing non-specialist audiences to visualize how runoff is intercepted, pre-separated, and filtered before storage for potential non-potable uses, reinforcing the real-world motivation of mitigating local flooding while recovering usable water.

**Figure 6:**
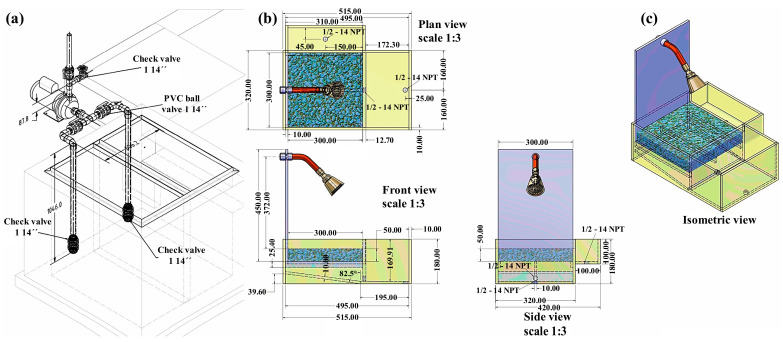
Technical schematic of the reduced-scale didactic prototype used for dissemination. (**a**) Hydraulic assembly showing the pump, threaded brass valve, PVC ball valve, check valves, and circulation line; (**b**) plan, front, and side views showing the acrylic compartment arrangement, pervious-concrete capture element, spray inlet, and hydraulic connection points; and (**c**) isometric view showing the spatial arrangement of the capture surface, transparent compartments, and inlet assembly. Dimensions are shown in mm, while threaded connections such as 1/2″-14 NPT and valve sizes of 1″ and 1 1/4″ correspond to nominal pipe/thread specifications in inches.

**Figure 7:**
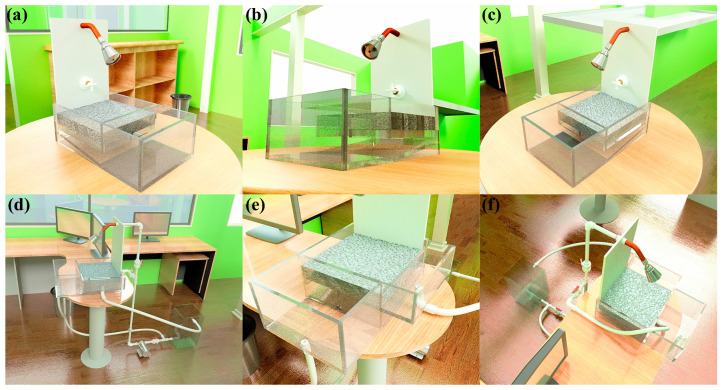
Constructed reduced-scale dissemination prototype and main components. Photographs show (**a**) a general view of the transparent acrylic body with the pervious-concrete capture section and spray head; (**b**) a side view highlighting the transparent compartment geometry and internal arrangement beneath the capture section; (**c**) an oblique view of the pervious-concrete capture section and adjacent compartment arrangement; (**d**) a wider view of the hydraulic connections and pump-assisted circulation line used to reproduce staged pretreatment; (**e**) an upper oblique view of the capture section, transparent compartments, and transfer/outlet tubing; and (**f**) an upper view of the capture section, spray inlet, circulation tubing, and hydraulic connections.

**Figure 8:**
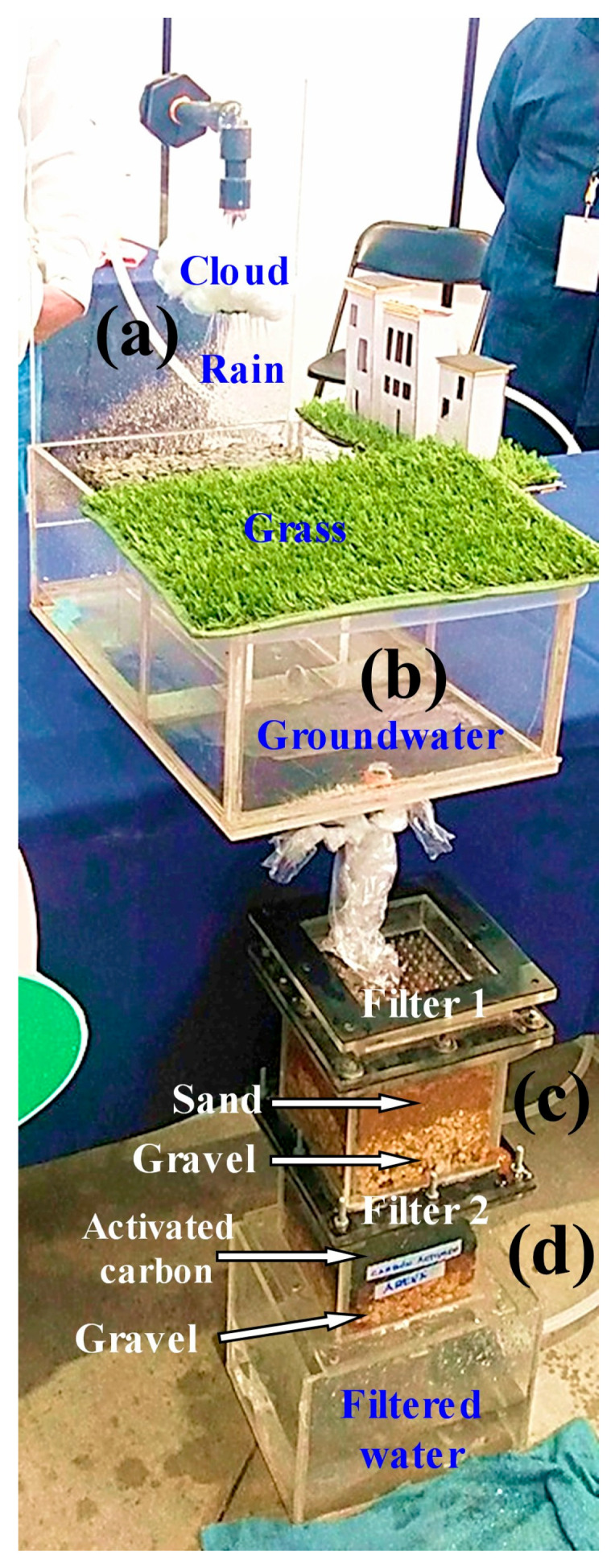
Representative demonstration sequence of the reduced-scale prototype. Photographs show (**a**) simulated rainfall over the pervious-concrete capture section, (**b**) visible water accumulation and transfer through the transparent compartments, (**c**) staged pretreatment prior to filtration, and (**d**) reduced-scale filtration through sand/gravel and activated carbon before collection of the filtered water.

For greater clarity, the dissemination material is presented as a separate visual sequence. Figure 6 provides the schematic and dimensional definitions of the reduced-scale didactic prototype. Figure 7 shows the constructed prototype and its main components. Figure 8 presents the representative demonstration sequence. Table 4 and Table 5 summarize the physicochemical gradients and calculated pretreatment efficiencies, respectively. Therefore, the water-quality results should be interpreted as compartment-level contributions rather than as a single black-box performance claim.

Limitations and next steps: (i) explicitly report the hydraulic loading rate (rainfall intensity/volume captured per event and residence times) to link performance to operating conditions, (ii) add a simple first-order mass balance/removal framework for turbidity/COD to separate sedimentation from media filtration, and (iii) include a minimum maintenance protocol (inspection frequency, activation of sediment removal and media replacement criteria) to support long-term reproducibility and transferability.

Figure 6 provides the schematic and dimensional definition of the dissemination model. The plan, front, side, and isometric views clarify the geometry and relative arrangement of the transparent acrylic compartments, the simulated capture zone containing the pervious concrete element, and the hydraulic connection points that govern flow direction. The inclusion of valve and check-valve elements in the assembly drawing is particularly valuable for reproducibility because it shows how the prototype can be operated in a controlled manner during demonstrations, enabling staged water transfers across compartments to emulate pretreatment logic at a reduced scale. In this sense, Figure 6 functions not only as an illustration but as a construction-ready roadmap for replication in other schools or community settings.

Figure 7 shows the constructed reduced-scale dissemination prototype and its main components. The photographs document the transparent acrylic body, the pervious-concrete capture section, the compartment arrangement, the hydraulic connections, the pump-assisted circulation line, the spray inlet, transfer tubing, and the reduced-scale filtration elements representing sand/gravel and activated-carbon polishing. Separating these structural views from the operation sequence improves readability and allows the physical configuration of the didactic prototype to be interpreted more clearly.

Figure 8 presents the representative demonstration sequence of the reduced-scale prototype. The photographs show simulated rainfall over the pervious-concrete capture section, visible water accumulation and transfer through the transparent compartments, staged pretreatment prior to filtration, and reduced-scale filtration through sand/gravel and activated carbon, followed by collection of the filtered water. This sequence illustrates how the prototype visually conveys the capture–pretreatment–filtration logic of the full-scale stormwater harvesting system for educational and dissemination purposes.

## 5. Conclusions

This work develops and field-deploys a decentralized stormwater harvesting system in which pervious concrete is deliberately reconfigured from a passive infiltration material into a controlled capture interface integrated within a staged treatment architecture. By incorporating an impermeable sublayer beneath the pervious concrete slab, the system forces hydraulic conveyance toward a defined treatment pathway, enabling runoff to be captured and managed rather than lost through uncontrolled infiltration.

The integration of a double-compartment pumping station introduces a staged pretreatment strategy that combines hydraulic buffering with sequential solids separation. The observed reductions in apparent color, turbidity, and COD across the compartments confirm that this configuration effectively conditions the runoff prior to downstream treatment. In this context, the system operates not only as a collection mechanism but also as a first barrier, reducing particulate loading and protecting subsequent filtration units from premature clogging.

The operational sequence, in which water is pumped from the second compartment after rainfall events and then filtered through granular and activated carbon, establishes a controlled, modular treatment process. This arrangement allows for temporal separation between capture and treatment, facilitating system control, maintenance, and adaptation to variable runoff conditions. The results obtained during the commissioning phase demonstrate the feasibility of this configuration under real field conditions at a school scale.

At the same time, the elevated pH, electrical conductivity, and total dissolved solids observed during initial operation highlight the influence of fresh cementitious materials and the need for system conditioning prior to long-term evaluation. These findings reinforce the importance of curing, flushing, and stabilization stages in systems that incorporate newly constructed concrete components.

It is important to note that this work primarily validates the capture and pretreatment stages of the system. The water-quality results reported correspond to intermediate conditions prior to final filtration and therefore do not constitute full verification of suitability for reuse. Additional evaluation of post-filtration water quality, including microbiological parameters, is required to establish compliance with non-potable reuse standards.

Future work should expand the validation framework in three directions. First, direct mechanical and durability testing or structural simulation of the pervious concrete capture slab should be conducted under repeated pedestrian and light-vehicle loading, including assessment of compressive/flexural performance, abrasion resistance, carbonation depth, and post-exposure changes associated with cementitious leaching and portlandite depletion. Second, post-filtration monitoring should include heavy metals and microbiological indicators, including total coliforms and *E. coli*, to define final reuse scenarios with greater certainty. Third, long-term performance should be evaluated under event-based maintenance conditions, including surface cleaning, sediment removal from the sump compartments, pump and valve inspection, backwashing or replacement of granular media, and replacement or regeneration of activated carbon when adsorption breakthrough is observed.

Overall, the system provides a low-infrastructure, replicable solution for decentralized stormwater management in institutional environments. Its main contribution lies in integrating hydraulic control and staged pretreatment into a single configuration, offering a practical pathway to reduce localized flooding while enabling the recovery and conditioning of stormwater for non-potable applications.

## Figures and Tables

**Table 1:** Permeable concrete mix matrix evaluated in laboratory tests. The contents of cement, coarse aggregate, water, and the waterproofing additive are reported as weight percentages (wt.%).

Aggregate Fraction	Cement (wt.%)	Coarse Aggregate (wt.%)	Water (wt.%)	Waterproofing Additive (wt.%)
Sieve No. 4 (4.75 mm)	16.58	77.58	5.78	0.07
Sieve No. 3/8 (9.50 mm)	16.32	77.94	5.68	0.07
3/8″ (9.525 mm)	16.17	78.14	5.63	0.06
1/2″ (12.7 mm)	15.76	78.66	5.52	0.06
5/8″ (15.875 mm)	15.51	79.01	5.42	0.06
3/4″ (19.05 mm)	15.21	79.41	5.32	0.06

**Table 2:** Recommended maintenance and consumable replacement criteria for the constructed stormwater harvesting system.

Component	Main Risk	Maintenance Action	Suggested Trigger
Pervious concrete Surface.	Surface clogging by sediment, leaves, and trash.	Sweeping, washing, and removal of coarse solids.	After rainfall events or when ponding is observed.
Impermeable sublayer/conveyance path.	Obstruction or leakage.	Visual inspection where accessible; confirmation of flow toward the sump.	During commissioning and after intense storms.
Sump Compartment 1.	Sediment accumulation.	Remove settled solids and standing water if needed.	Visible sediment or reduced hydraulic transfer
Sump Compartment 2.	Fine solids and residual water accumulation.	Pump-out, flushing, sediment removal.	After rainfall events or before long inactive periods.
Pump and valves.	Loss of suction, blockage, leakage.	Check valve positions, flush lines, inspect pump.	Each operation cycle.
Sand/gravel filter.	Clogging and turbidity breakthrough.	Backwash, clean, or replace media.	Flow reduction or turbidity increase.
Activated carbon filter.	Adsorption exhaustion.	Replace or regenerate activated carbon.	Color/odor/COD breakthrough; approximately every two years under continuous use.

**Table 3:** Construction and commissioning milestones for the school-scale system (photographic record).

1. Layout and placement in an educational institution.	2. Beginning of excavations.	3. Material hauling, concrete slab shoring, and pumping sump.
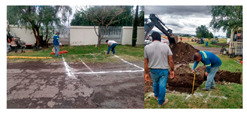	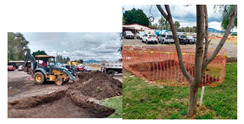	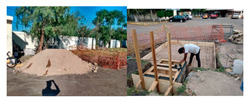
4. Shoring and casting of filters.	5. Assembly of filter pipes and last rains of 2023.	6. Demolding of the shoring and filters.
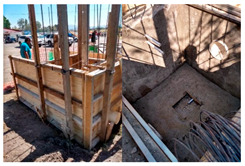	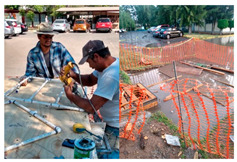	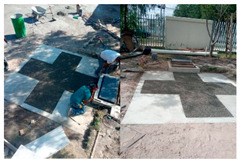
7. Placement of sump caps.	8. Placement of flanges in filters.	9. Placement of the pump and water sampling in the last rains of 2023.
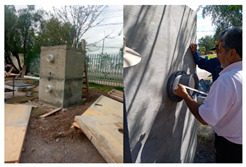	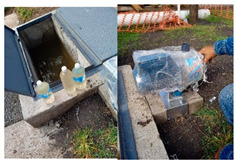	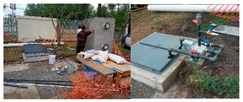
10. Installation of pipes.	11. Pervious concrete samples for laboratory analysis and filling of filter media.	12. Filling filters with filter media and hydraulic testing.
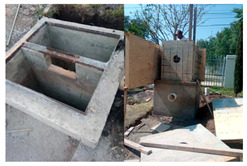	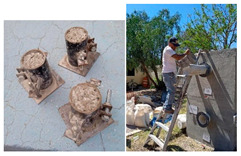	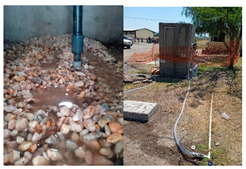
13. Leaks in filters and alternative repair of stainless steel tank.	14. Filter repair using two stainless steel tanks and hydraulic testing of the same	15. Hydraulic tests completed.
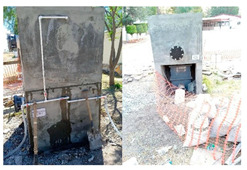	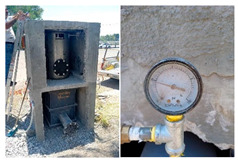	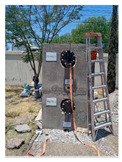

**Table 4:** Physicochemical indicators of runoff quality and staged pretreatment at sampling: influent reference, sump Compartment 1, and sump Compartment 2. The data were used to infer sedimentation/clarification trends and ionic load prior to sand/gravel filtration and activated-carbon polishing. (ND = not determined).

Parameter	Influent Reference (Puddle)	Compartment 1	Compartment 2
pH	8.44	11.0	9.0
ORP	−87.9 mV	ND	ND
Electrical conductivity	0.129 mS/cm	7.78 mS/cm	1.963 mS/cm
Total Dissolved Solids	71.71 mg/L	3900 mg/L	985 mg/L
Total hardness	120 mg/L	ND	ND
Alkalinity	120 mg/L	ND	ND
Colour	1510 U pt/Co	221 U pt/Co	59 U pt/Co
Sulphates	0 mg/L	ND	ND
Turbidity	ND	46.8 NTU	12.91 NTU
COD	ND	30 to 35 mg/L	15 to 18 mg/L

**Table 5:** Approximate pretreatment efficiencies calculated between Compartment 1 and Compartment 2 of the double-compartment sump.

Parameter	Compartment 1	Compartment 2	Approximate Reduction
Apparent color	221 Pt-Co	59 Pt-Co	73.3%
Turbidity	46.8 NTU	12.91 NTU	72.4%
COD	30–35 mg·L^−1^	15–18 mg·L^−1^	~40–57%

**Table 6:** Qualitative comparison between conventional storm-drainage infrastructure and the proposed decentralized harvesting system, considering cost drivers, maintenance needs, and functional outputs.

Aspect	Conventional Drainage Solution	Proposed Decentralized Harvesting System
Main objective	Rapid conveyance and discharge of runoff.	Local capture, pretreatment, storage, and non-potable reuse.
Main infrastructure	Inlets, underground pipes, excavation, and discharge route.	Pervious-concrete slab, impermeable sublayer, double-compartment sump, pump, sand/gravel filter, activated-carbon filter, 5000 L tank.
Main cost drivers	Excavation depth, pipe length, inlet structures, and connection to the existing drainage network.	Capture slab, liner, sump construction, pump, filters, storage tank, filter media.
Water recovery	Usually not included.	Included through storage for non-potable use.
Pretreatment function	Generally absent or limited to inlet sediment capture.	Staged sump clarification followed by sand/gravel and activated-carbon polishing.
Maintenance needs	Inlet cleaning, pipe inspection, and blockage removal.	Surface cleaning, sump sediment removal, pump inspection, filter backwashing, and activated-carbon replacement.
Practical advantage	Efficient runoff removal from the site.	Reduced ponding, plus local reuse and educational value.
Main limitation	No local water reuse and dependence on drainage network capacity.	Requires operation, maintenance, and post-filtration validation.
Recommended cost metric	Cost per drained area or pipe length.	Cost per treated catchment area, cost per storage volume, and maintenance cost per rainfall season.

## Data Availability

The original contributions presented in this study are included in the article. Further inquiries can be directed to the corresponding authors.
